# Low employment and low willingness of being reemployed in Chinese working-age maintained hemodialysis patients

**DOI:** 10.1080/0886022X.2017.1361834

**Published:** 2017-08-14

**Authors:** Bihong Huang, Bihong Lai, Ling Xu, Ying Wang, Yanpei Cao, Ping Yan, Jing Chen

**Affiliations:** a Department of Nursing, Huashan Hospital Fudan University, Shanghai, China;; b Department of Nursing, Pudong Hospital, Fudan University, Shanghai, China;; c Department of Nursing, Shanghai Fifth Hospital, Fudan University, Shanghai, China;; d Department of Nursing, The 455th Hospital of PLA, Shanghai, China;; e Department of Cardiology, Huashan Hospital Fudan University, Shanghai, China;; f Department of Nephrology, Huashan Hospital Fudan University, Shanghai, China

**Keywords:** Employment, hemodialysis, social support

## Abstract

**Aim:** Returning to society plays an important role in improving the quality of life in maintenance hemodialysis (MHD) patients, and retention of employment is one of the core enablers. The study is to assess the employment status and to determine the variables for unemployment in Chinese MHD patients.

**Methods:** Prevalent MHD patients from four dialysis centers in Shanghai China were enrolled. We assessed patients’ employment status, current social functioning, hemodialysis modality, annual income and general health condition. Among current unemployed working-age patients, the reasons of quitting jobs and willingness of being reemployed were evaluated.

**Results:** A total of 231 patients were studied, among which 114 patients were unemployed 1 year before hemodialysis. Among 117 employed patients, 16 patients quitted jobs before dialysis inception, while 49 patients quitted jobs at the initiation of HD, and 26 patients followed after a few months’ HD. The main reasons for ceasing employment were physical insufficiency, conflict between dialysis and work schedules, lack of support from employers and resistance from family members. Among the 166 patients who were in their working age, 26 patients were employed. The unemployed patients had the characters of elder age, lower education level, higher annual family income, higher female ratio, lower blood flow, lower physical functioning, and social functioning and lower frequency of weekend hemodialysis and HDF/HF. Among the 140 unemployed patients, only 47 patients had the willingness of being reemployed. Their unemployment status was positively associated with elder age ((OR) 3.13, 95% CI, 1.08–9.1), lower education level ((OR) 1.97, 95% CI, 1.05–5.92), and higher family income ((OR) 7.75, 95% CI, 2.49–24.14).

**Conclusion:** Ratio of employment and willingness of being reemployed was low in MHD working-age patients. Lack of social and family’s support also hampered patient’s returning to society except for the HD treatment quality.

## Introduction

The prevalence of chronic kidney disease is high in China [1,2]. The use of dialysis for patients reaching end-stage renal disease (ESRD) is rapidly increasing and approaching that of USA [3,4]. Till the end of 2015, the number of dialysis patients is over 448,000 (385,000 for hemodialysis (HD) and 63,000 for peritoneal dialysis (PD)), which is far behind the estimated 1 million ESRD patients who need dialysis in current China [5].

Although ESRD patients value longer living, quality of life (QoL) is a key prognostic factor for them and has been taken into the most important consideration for patient management, once the fundamentals and complexities of clinical management have been addressed [6]. Many factors can affect the Qol in HD patients. One critical factor is occupational status which also contributes a lot to rehabilitation as well as the improvement of economic status and emotional state by recovering self-esteem and various capabilities [7–9].

According to the recent annual report of Chinese Renal Data System, over 60% of all prevalent or incident HD patients are in the working age bracket of 18–60 years in China, and the retirement age is predicted to increase in coming years [5]. What’s more, the new ESRD patients who accept dialysis are boosting as the result of improvement of reimbursement policy in China. And the HD modality predominates over 85% among dialysis patients. As such, we believe that it is important and urgently necessary to assess the current employment status in HD patients and to analyze the reasons of impeding their employment, especially if they are actively employed or wish to be, in order to improve the quality of hemodialysis. However, there are limited data on this topic in Chinese hemodialysis patients. Thus, a pilot study on the employment status was carried out in multi-centers in Shanghai.

## Methods

### Study design and setting

A patient survey was used in this study for participants from four hemodialysis centers in Shanghai China. The represented centers in this study were selected considering the academic level and area. Two teaching hospitals affiliated with Fudan University (one from urban area and the other one from rural area) and two non-teaching hospitals (also one urban area and the other one from rural area) were selected among the total 66 HD centers in Shanghai. This study protocol was approved by the Ethics Committee of Huashan Hospital Fudan University. A formal written consent was obtained from all participants by research nurses. And the lab data was collected from hospital’s data base by nurses manually. The survey was conducted from July 2015, with survey data lock at the end of November 2015.

### Participants and variables

All HD patients in four HD centers were screened following the main inclusion criteria: (1) patients were on MHD over 3 months. (2) Patient’s age was between 18 and 60 years old when inception of hemodialysis. And the main exclusion criteria were as follows: (1) patients were unwilling to participate. (2) Patients had medical history of psychiatric disorders. (3) Patients initiated dialysis with PD over 3 months.

The primary outcome in our study was current employment status. We recorded the nature of employment and modeled employment as a single binary variable, and included household labor as a type of employment. Variables including demographic data, annual family income level, HD modality and main HD parameters, lab data, erythropoietin usage, and SF-36 score were collected to analyze the association with employment status. The secondary outcome was the employment status one year prior to HD inception and the willingness of being reemployed among the current unemployed HD patients in working age bracket.

### Description of survey instrument

The survey instrument was developed by investigators at Huashan Hospital Fudan University composing of the Chinese version of the SF-36 (version 2) and supplementary questions. The Chinese version of the SF-36v2 composed of a single item of health transition (HT) and 35 items that can be divided into eight subscales, namely physical function (PF), limitations due to physical health problems (role-physical, RP), bodily pain (BP), general health (GH), vitality (VT), social functioning (SF), limitations due to emotional health problems (role-emotional), and mental health (MH). Supplementary questions focused on two major areas: employment status before and after the inception of regular dialysis treatment (e.g., select your current employment and that at the time of one year prior to dialysis, fill in the time that the patient quitted job and the job type including company employee, self-employed, part-time job, household labor), and the socioeconomic status of participants (e.g., select your current annual family income and the co-pay of HD).

The survey was completed by HD nurses. A group discussion on how to carry out the survey was held before this study. Pre-testing was also conducted through a limited pilot at Huashan Hospital Fudan University to make sure that the questions were valid in providing accurate concept and the patients could correctly understand them.

### Data analysis

Descriptive analyses were performed using parametric (*t*-test) or non-parametric (Mann–Whitney *U*-test) tests as appropriate. Chi-square test was applied for proportion comparison. Associations between the outcomes and predictors were explored using univariate and multivariate logistic regression analyses. All data were expressed as the means ± SD, unless otherwise indicated. Statistical significance was set at *p* < .05. All analyses were performed by using SPSS version 16.0 (SPSS Inc., Chicago, IL).

## Results

### Descriptive data

A total of 231 patients were enrolled. Sixty-five patients have reached the age of retirement when doing this survey. The remaining 166 patients in the working age bracket were regarded as a subgroup for further analysis. Patient characteristics are shown in Tables 1 and 2, by employment status. In the subgroup, patients who were employed were shown to be younger, better-educated and had higher blood flow, higher frequency of weekend shift, higher medium-large molecule elimination hemodialysis modality (hemoperfusion or High flux HD or hemodiafiltration) and higher annual family income.

**Table 1.** Characteristics of patients one year prior to dialysis.

Variable	Overall	Employed^a^	Non-employed	*p* Value
*n* (%)	231	117 (50.65%)	114 (49.35%)	<.05
Age (years)	43.94 ± 9.69	42.33 ± 9.08	45.55 ± 10.15	<.05
Gender (F/M)	91/140	39/78	52/62	<.05
Diabetic nephropathy	28 (12.1%)	15 (12.8%)	13 (11.4%)	.76
Educational level				<.05
College and above	37 (16%)	31 (26.5%)	6 (5.3%)	
High middle school	71 (30.7%)	33 (28.2%)	38 (33.3%)	
Middle school	89 (38.5%)	42 (35.9%)	47 (41.2%)	
Primary school and below	34 (14.7%)	11 (9.4%)	23 (20.2%)	
With medical insurance	224 (97%)	114 (97.4%)	110 (96.5%)	.78

a Including full time, part time job, and house labor.

**Table 2.** Characteristics of prevalent patients who are in working age^a^.

Variable	Overall	Employed	Non-employed	*p* Value
*n* (%)	166	26 (15.7%)	140 (84.3%)	<.05
Age (year)	48.46 ± 8.4	44.55 ± 8.89	49.41 ± 7.4	<.05
Gender (F/M)	60/106	7/19	53/87	<.05
Diabetic nephropathy	23 (13.9%)	4 (15.4%)	19 (13.6%)	.81
Cancer	2	2	0	NA
Educational level				<.05
College and above	25	15	10	
High middle school	59	5	54	
Junior middle school	62	6	56	
Primary school and below	20	0	20	
With medical insurance	156 (93.98%)	22 (84.62%)	134 (95.71%)	<.05
Annual family income				<.05
<50,000 RMB^b^	93 (56%)	5 (19.2%)	88 (62.9%)	
50,000–100,000 RMB	42 (25.3%)	4 (15.4%)	38 (27.1%)	
100,000–150,000 RMB	23 (13.9)	12 (46.2%)	11 (7.9%)	
>150,000 RMB	8 (4.8%)	5 (19.2%)	3 (2.1%)	
Hemodialysis vintage	7.12 ± 5.11	5.6 ± 4.33	7.46 ± 5.6	.09
Blood flow (ml/min)	224 ± 19	236 ± 24	221 ± 18	<.05
Weekly HD^c^ frequency	2.89 ± 0.33	2.87 ± 0.33	2.91 ± 0.28	.92
With treatment on weekend^d^	79 (47.59%)	17 (65.38%)	62 (44.29%)	<.05
HD with AVF^e^	155 (93.37%)	25 (96.15%)	130 (92.77%)	.28
HP or HFHD or HDF weekly^f^ frequency				<.05
0	65 (39.16%)	8 (30.77%)	57 (40.71%)	
<1	20 (12.05%)	3 (11.54%)	17 (11.14%)	
≥1	81 (48.8%)	15 (57.69%)	66 (47.14%)	
Hb (g/L)	107.78 ± 20.78	106.73 ± 21.82	108.14 ± 20.45	.39
P (mmol/L)	2.108 ± 0.625	2.19 ± 0.60	2.100 ± 0.624	.44
Ca (mmol/L)	2.272 ± 0.314	2.30 ± 0.27	2.272 ± 0.327	.63
PTH (pg/mL)	381.88 ± 502.05	429.79 ± 509.70	367.05 ± 500.75	.56
ALB (g/dL)	41.14 ± 5.95	41.50 ± 4.17	41.11 ± 6.53	.74
Pre-ALB (g/dL)	340.31 ± 95.44	338.30 ± 92.80	344.41 ± 96.90	.77
Kt/V	1.366 ± 0.338	1.37 ± 0.316	1.365 ± 0.345	.96
URR(%)	66.10 ± 8.807	66.80 ± 9.05	65.87 ± 8.71	.73

a Working age: 18–60 years old.

b RMB: Renminbi (Chinese Yuan).

c HD: hemodialysis.

d With treatment on weekend: one HD treatment was done at Saturday.

e AVF: arteriovenous fistula.

f HP: hemoperfusion; HFHD: high flux HD; HDF: hemodiafiltration.

### Employment status 1 year prior to dialysis and changes after dialysis inception

In this cohort, 117 (50.65%) participants were employed one year prior to dialysis, while 114 (49.35%) were unemployed as shown in Table 1. Sixteen patients quitted jobs before dialysis inception. Seventy-five patients gave up their employment after dialysis inception, among which 49 patients quitted jobs immediately after inception of dialysis, while 26 patients stopped working an average 12 month later. Among the current employed 26 patients, only eight patients were on full time job. No patient was reemployed in the unemployed group.

The reasons for quitting job were also surveyed in this cohort. Most patients (87%) stated that they did not feel well enough to work, while 55% claimed that the dialysis time was a stumbling block. Other reasons given were lack of support/acceptance from employers (43%) and resistance from family members (31%).

### QoL assessment from SF-36 responses by employment status in the subgroup

Overall, social functioning of the patients at the time of the survey was reported to be low, indicating severe and frequent interference with normal social activities due to their physical and/or emotional problems (Table 3). There were no differences in psychometric constructs by employment status except that physical functioning scores and social functioning scores were higher in the employed group (76.05 ± 17.40 versus 69.42 ± 20.33; 75.48 ± 18.35 versus 66.42 ± 24.07).

**Table 3.** QoL assessment from SF-36 responses by employment status in the subgroup.

Variable	Overall (*n* = 166)	Employed (*n* = 26)	Non-employed (*n* = 140)	*p* Value
Physical functioning	71.78 ± 19.49	76.05 ± 17.40	69.42 ± 20.33	.03
Role physical	40.24 ± 40.24	48.21 ± 39.49	36.54 ± 40.24	.1
Bodily pain	74.68 ± 20.52	75.76 ± 18.51	74.55 ± 20.90	.74
General health	43.02 ± 19.52	45.98 ± 20.28	42.37 ± 19.42	.31
Vitality	61.27 ± 20.04	61.95 ± 20.42	60.84 ± 20.14	.76
Social functioning	69.37 ± 22.99	75.48 ± 18.35	66.42 ± 24.07	.04
Role emotional	53.13 ± 44.33	60.32 ± 41.30	50.85 ± 45.04	.23
Mental health	69.82 ± 19.28	72.10 ± 16.63	68.71 ± 20.14	.33

### Willingness of being reemployed in the subgroup

The willingness of being reemployed in the subgroup is surprisingly low. Among the 140 unemployed patients, only 47 (33.6%) patients reported that they would be interested in returning to work. There was no difference between two groups except that the female ratio and educational level were higher in the patients who showed willingness to be reemployed (data not shown).

### Independent predictors of current unemployment status in the subgroup

In the univariate model, increasing age, lower educational level, higher annual family income, no weekend treatment and lower SF36-role physical score were associated with loss of employment. To avoid model overfitting and the possible effects of confounding factors, all variables with *p* values less than 0.2 under the univariate analysis were considered as potential predictors in the multivariate analysis. In the multivariate model, increasing age, lower educational level and higher annual income were identified as independent risk factors for loss of employment (Table 4).

**Table 4.** Factors affecting current unemployment status.

	Univariate model		Multivariate model	
	Odds ratio (95% CI)	*p* Value	Odds ratio (95% CI)	*p* Value
Female gender (female versus male)	1.65 (0.651–4.197)	.29		
Older age (≥50 versus <50)	1.104 (1.014–1.116)	.012	3.134 (1.079–9.1)	.036
Teaching hospital (yes versus no)	0.702 (0.273–1.924)	.375		
Center location (urban versus rural)	0.828 (0.479–2.106)	.522		
Primary disease (DM versus others)	2.198 (0.709–6.809)	.17	0.373 (0.094–1.476)	.16
Educational level(below high middle school versus others)	27.879 (2.678–290.25)	.005	1.971 (1.057–5.916)	.026
Higher annual family income (≥100,000 RMB versus others)	0.316 (0.193–0.516)	<.001	7.749 (2.487–24.143)	<.001
Medical insurance(with versus without)	0.641 (0.177–2.314)	.497		
Middle-large molecule elimination modality^a^ (with versus without)	1.545 (0.629–3.295)	.343		
Middle-large molecule elimination modality (≥1 per week versus others)	0.722 (0.312–1.671)	.447		
Weekend dialysis(without versus with)	1.084 (1.006–1.164)	.01	1.924 (0.653–5.668)	.235
SF36-Physical Functioning (<75 versus ≥75)	0.981 (0.959–1.003)	.088	0.456 (0.144–1.451)	.811
SF36-Role Physical (<25 versus ≥25)	0.988 (0.978–0.998)	.02	1.091 (0.35–3.4)	.184
EPO usage (yes versus no)	0.574 (0.069–4.743)	.606		
Hemoglobin	1.472 (0.469–4.617)	.508		
Albumin	1 (0.979–1.022)	.968		

a Including high flux HD, hemoperfusion, and hemodiafiltration.

## Discussion

Only a small proportion of ESRD patients are employed at the start of dialysis compared with the general population [9–11]. Staying on job can benefit the patients in many ways such as a source of social support, a higher quality of life, increased self-esteem, more stable or higher financial situation. Moreover, maintenance of work is also important for healthy society in preventing loss of production [10,12,13].

The present study confirms earlier findings that ratio of employment was low in MHD working-age patients [8,10,12,14,15]. In our study, only 117 (50.65%) were employed one year before dialysis inception, and additional 16 patients quitted jobs before dialysis. Inception of hemodialysis seemed to be the last straw that broke the camel’s back in most cases. After HD began, only 26 (11.26%) patients kept on working. Moreover, we reported the willingness of being employed in Chinese MHD patients, showing that only 33.6% of the patients had the intention to return to work, which was also similar to other report [16]. It was relatively low and might indicate the great barriers that the patients were facing.

Many possible factors infusing employment status were reported such as age, gender, education levels, lifestyle, dialysis modality, medical insurance, serum albumin, anemia, physical and psychological functioning, disease etiology(diabetes), availability of late-shift dialysis, training, and high-frequency hemodialysis [9,10,17–21]. However, in our study, we observed that only increasing age, lower educational level, and higher annual income were identified as independent risk factors for loss of employment. While patient characteristics such as level of education, elder age, and occupational status before dialysis remained fixed, it is axiomatic that facility-level characteristics could be modified in efforts to increase patients’ opportunity to be employed [20].

Many reports stated that in center HD (ICHD) was less conductive to employment, as a result of the consumed time of HD and traveling between home and dialysis facility as well as the post-dialysis ‘downtime’ [8,14,17,22]. In the present study, the main reasons for quitting jobs are also the physical function and dialysis time in our population. Thus, promoting gainful employment among ESRD patients continues to be a quality improvement need as well as providing convenient dialysis time for patients [20,23–25]. Surprisingly in our study, resistance from family member and lack of acceptance by employers were another two main barriers to employment. That may indicate that patients including their family members, caregivers and even the social employers should also be educated about treatment choices and therapeutic goals for kidney failure [6,26]. What’s more, social support programs such as spiritual care, employment counseling or vocational rehabilitation may lower some of the barriers to employment and can help individuals with the greatest potential for workforce participation [27–30].

We acknowledge some potential limitations in our study. First, there are possibilities of recall bias, and residual confounding from lack of information collected on social supports and family situation. Second, the present study was a retrospective analysis of prevalent patients, not including the cases of early deaths after dialysis inception. Third, our data did not include some comorbid condition that may affect ability to work, such as infections and cardiovascular diseases.

In summary, patients in working age are at significant risk of losing their employment, especially after the introduction of chronic dialysis treatment. Willingness of returning to work in this population is relatively low. Since preserving socialization and socioeconomic status of patients is one of the core objects, how to help working-age patients keep their jobs should be taken into serious consideration when developing health service policy or quality control program among ESRD population. In addition, employment was hampered by employer and family members’ conception, thus there is urgent need to raise the awareness of treatment goal in public for this population.

## Ethical approval

‘All procedures performed in studies involving human participants were in accordance with the ethical standards of the institutional and/or national research committee and with the 1964 Helsinki declaration and its later amendments or comparable ethical standards’. This study protocol was approved by the Ethics Committee of Huashan Hospital Fudan University.

*Informed consent*: ‘Informed consent was obtained from all individual participants included in the study’.
